# MiR-21 in Extracellular Vesicles Leads to Neurotoxicity via TLR7 Signaling in SIV Neurological Disease

**DOI:** 10.1371/journal.ppat.1005032

**Published:** 2015-07-08

**Authors:** Sowmya V. Yelamanchili, Benjamin G. Lamberty, Deborah A. Rennard, Brenda M. Morsey, Colleen G. Hochfelder, Brittney M. Meays, Efrat Levy, Howard S. Fox

**Affiliations:** 1 Department of Pharmacology and Experimental Neuroscience, University of Nebraska Medical Center, Omaha, Nebraska, United States of America; 2 Nathan S. Kline Institute, Orangeburg, New York, Departments of Pathology, Psychiatry, and Biochemistry and Molecular Pharmacology, New York University Langone Medical Center, New York, New York, United States of America; National Institutes of Health, UNITED STATES

## Abstract

Recent studies have found that extracellular vesicles (EVs) play an important role in normal and disease processes. In the present study, we isolated and characterized EVs from the brains of rhesus macaques, both with and without simian immunodeficiency virus (SIV) induced central nervous system (CNS) disease. Small RNA sequencing revealed increased miR-21 levels in EVs from SIV encephalitic (SIVE) brains. In situ hybridization revealed increased miR-21 expression in neurons and macrophage/microglial cells/nodules during SIV induced CNS disease. In vitro culture of macrophages revealed that miR-21 is released into EVs and is neurotoxic when compared to EVs derived from miR-21^-/-^ knockout animals. A mutation of the sequence within miR-21, predicted to bind TLR7, eliminates this neurotoxicity. Indeed miR-21 in EV activates TLR7 in a reporter cell line, and the neurotoxicity is dependent upon TLR7, as neurons isolated from TLR7^-/-^ knockout mice are protected from neurotoxicity. Further, we show that EVs isolated from the brains of monkeys with SIV induced CNS disease activates TLR7 and were neurotoxic when compared to EVs from control animals. Finally, we show that EV-miR-21 induced neurotoxicity was unaffected by apoptosis inhibition but could be prevented by a necroptosis inhibitor, necrostatin-1, highlighting the actions of this pathway in a growing number of CNS disorders.

## Author Summary

HIV associated neurocognitive disorder (HAND) are neurological disorders caused due to the entry of HIV infection in the brain. HIV-1 does not directly infect central or peripheral neurons, however, virus-infected cells of the monocyte/macrophage lineage maintain a low-level HIV infection in the CNS. "Indirect effects" of macrophage activation–such as dysregulation of cytokines and chemokines, free-radical (oxidative stress) injury, and secretion of soluble factors that are potently neurotoxic–have been implicated as effectors of nervous system injury in HIV. Here, we report that extracellular vesicles released from macrophages can enhance neurotoxicity. Using a nonhuman primate model of HAND, simian immunodeficiency virus encephalitis (SIVE), we find that exosomes isolated from SIVE brains contain,microRNAs, including miR-21, that can serve as ligands to the key immune regulatory receptors, toll-like receptors, and can elicit neurotoxicity. We provide in vitro evidence for such an effect, and that the toxicity can be mediated by necroptosis. Thus, our study provides insights into other potential neurotoxic mechanisms by which HIV infection in the brain could harm neuronal health.

## Introduction

HIV-associated neurocognitive disorder (HAND) is a central nervous system (CNS) associated neurological disease where neurodegeneration is a consequence of CNS inflammation. The pathological characteristics of the most extreme form of this disease include astrogliosis, microgliosis, presence of multinucleated giant cells, and loss of dendrites and synapses [1–3], collectively termed HIV encephalitis (HIVE). These features are recapitulated in its nonhuman primate equivalent rhesus macaque model, simian immunodeficiency virus encephalitis (SIVE) [4]. In the CNS, HIV primarily infects microglia and macrophages but not the neurons. However, inflammatory molecules, as well as HIV gene products that are released from infected cells, have damaging affects on neurons [5–8]. Previously, others and we identified that SIV/HIV infection upregulated microRNAs (miRNAs) in macaque and human brains [9–11]. These studies have shown that upregulation of miRNAs can also lead to neuronal dysfunction by targeting crucial genes and by repressing their expression in the CNS. Further, we also identified that some of these miRNAs can be released extracellularly in extracellular vesicles (EVs) [12]. EVs are small membrane-bound structures. They play a significant role in cell-cell communication [13–16], in progression of cancer [17] and in viral infections [18–20]. In the brain, astrocytes [21], microglia [22] and neurons [23] have been shown to release EVs such as exosomes under physiological conditions. There is growing evidence for intercellular EV transfer within the CNS. EVs have been repeatedly discussed as potential carriers in the dissemination of disease pathology in neurodegenerative disorders, as they harbor proteins and RNA that can be transferred from the originating cell to a target cell [24].

We have previously identified that miR-21 is significantly upregulated during SIV/HIV infection in the brain [11]. Thus, we hypothesized that miR-21 may be present within EVs during SIV/HIV associated neuroinflammation and therefore, can be damaging to neurons. Intriguingly, a recent study indicated that certain extracellular miRNAs could bind to toll-like receptors (TLRs) in neurons and cause neurodegeneration [25]. These miRNAs had a G/U rich region capable of activating TLR7/TLR8. Interestingly, miR-21 is one such miRNA.

The overall goal of this study was to investigate whether miR-21 was significantly enriched in EVs in SIVE pathogenesis and if such an increase induces deleterious signaling pathways downstream. Here, for the first time, we report the miRNA profiling of EVs from the brain. We find that miR-21 is increased in EVs during SIVE pathogenesis and that it is deleterious to neurons by activating TLR7 dependent downstream cell death pathways. Hence, our data provide insight into the evolving EV-biology field and further expands our knowledge on understanding the molecular mechanism underlying the cause for neuronal damage during SIV/HIV-infection of the brain.

## Results

### MiR-21 expression is significantly upregulated in extracellular vesicles isolated from the SIVE macaque brains

**Fig 1:**
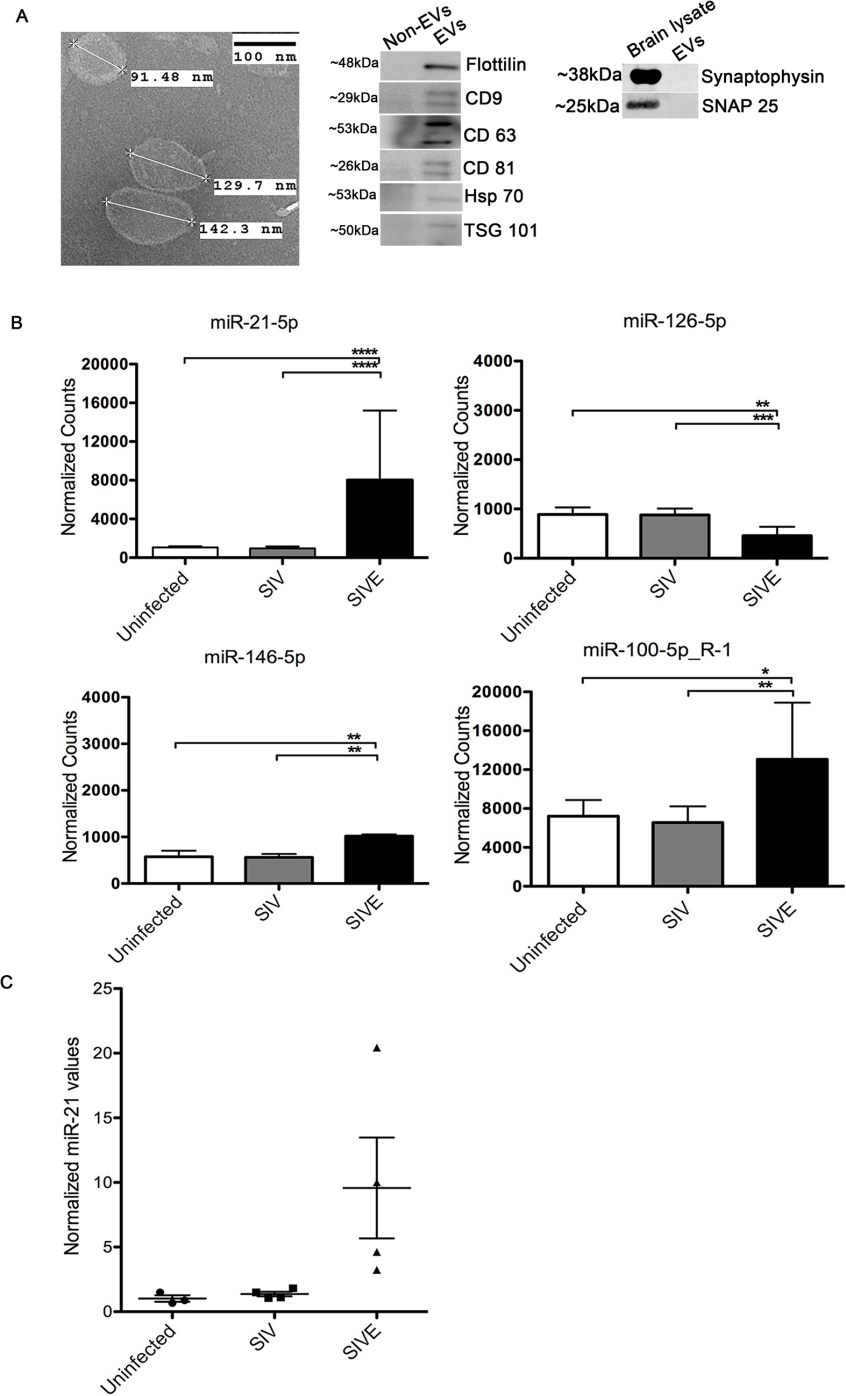
Isolation and characterization of brain derived EVs. **(A)** Left, Electron microscopic (TEM) morphological analysis of EVs derived from uninfected (control) macaque brain. EVs show a size range from 100–150 nm. Scale bar = 100 nm. Right, Western blots for flotillin, CD9, CD63, CD81, HSP70, TSG101 markers for EVs. Non-EV fractions from sucrose gradients were used as negative controls for the EV proteins, brain lysates were used as positive controls for the synaptic proteins. **(B)** Small RNA sequencing performed on RNA isolated from uninfected, SIV and SIVE brains. Analysis revealed significantly increased expression of miR-21-5p, miR-100-5p and miR-146-5p, and decreased expression miR-126-5p, in SIVE. Error bars = SEM; * *P* <0.05, ** *P* <0.01*** *P* <0.001, **** *P* < 0.0001; ANOVA with Tukey’s post-hoc test. **(C)** qRT-PCR validation of miR-21 expression in EVs. Relative quantification was performed based on a standard curve. Statistical analyses were performed on log-transformed values. One-way ANOVA showed p = 0.0024 with Tukey’s <0.01 for uninfected vs. SIVE, and SIV vs. SIVE.

Previously, we determined that miR-21 is upregulated in SIVE and HIVE [11]. Recent studies reported that certain miRNAs such as miR-21, if present extracellulary or in extracellular vesicles (EVs) could trigger TLR signaling pathways by acting as a ligand leading to cell injury [25–27]. Hence, we questioned whether miR-21 in association with EVs in SIVE neuropathology and whether this EV miR-21 (EV-miR-21) causes neuronal damage. EVs were isolated from SIVE and uninfected macaques brain regions using a sucrose gradient protocol [28]. Transmission electron microscopy (TEM) was used to characterize the EVs. The results revealed a size of ~100–150 nm with an appearance (cup-like) of vesicles that were previously described as exosomes (Fig 1A, left). Western blotting confirmed the presence of proteins associated with EVs: Flottilin, CD9. CD63, CD81, HSP70 and TSG101 (Fig 1A, right).

Next, we extracted RNA from EVs, and small RNA sequencing was conducted. The results revealed that miR-21 was significantly upregulated in EVs derived from the SIVE brain samples when compared to uninfected animals, as well as to SIV infected animals that did not have CNS disease (Fig 1B and S1 Table). Additionally, we also found two other miRNAs to increase at much lower levels of change and significance, miR-100-5p and miR-146-5p, and one miRNA to be decreased, miR-126-5p. The change in expression of miR-21 was then validated by quantitative real time polymerase chain reaction (qRT-PCR) on the EV samples for miR-21, revealing significantly elevated expression of miR-21 in SIVE samples (Fig 1C).

### Increased expression of miR-21 is seen in macrophage/microglial cells in SIVE brain tissues

**Fig 2:**
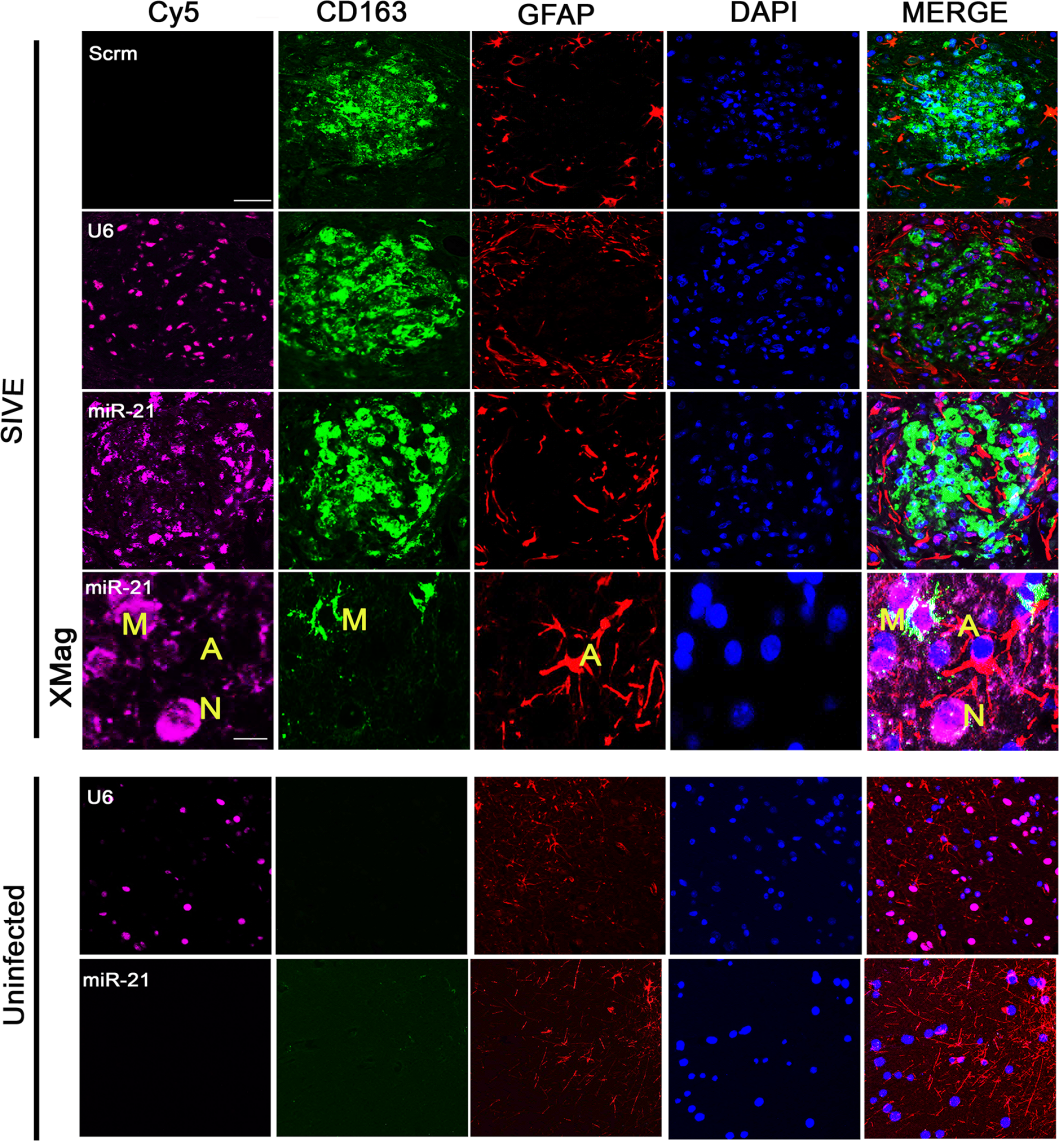
Combined FISH and IF for miR-21, CD163 and GFAP. In SIVE brain sections containing macrophage/microglial nodules, miR-21 (magenta) partially colocalized with the macrophage/microglia marker CD163 (green). No colocalization was observed with GFAP (red), an astrocyte marker. DAPI (blue) was used to label nuclei; a scrambled miRNA probe (Scrm) was used as negative control for hybridization; and U6 probe (a non-coding snRNA) was used as a positive control. Scale bars = 20 μm for all panels except 5 μm for SIVE-Mag panels.

Our initial studies found that in SIVE miR-21 is upregulated in neurons [11]. Trans migration of cargo from EVs has been shown to enter neurons from non-neuronal cells such as macrophages, microglia and astrocytes [16]. During HIV and SIV infection, macrophages infiltrate the brain, and activated macrophages as well as microglia and astrocytes are found. In order to examine whether such non-neuronal cells in the brain express miR-21 during infection, we performed fluorescence in situ hybridization (FISH) coupled with immunofluorescent (IF) labeling on brain tissue sections of SIVE and uninfected macaques. As a positive control, U6, a noncoding snRNA, showed abundant signals in most cells in the tissue; as a negative control, a scrambled miRNA probe did not show any hybridization in these sections. Interestingly, miR-21 signal was seen in CD163 positive macrophages/activated microglial cells and cells with the phenotypic appearance of neurons, whereas minimal signaling is seen in GFAP positive astrocytes (Fig 2, SIVE-Mag panel). In uninfected controls, miR-21 expression was below the detection limit, although U6 could still be detected (Fig 2, Uninfected panels). Therefore, it is possible that during infection macrophages could secrete EVs containing miR-21 that could then affect neurons. Given the prime role of macrophages in neuropathogenesis of HIV/SIV and the presence of miR-21 in macrophages in the infected brain, we used macrophages as the cellular model for EV release in our experiments.

### Presence of miR-21 in synthetic EVs renders neurotoxicity

**Fig 3:**
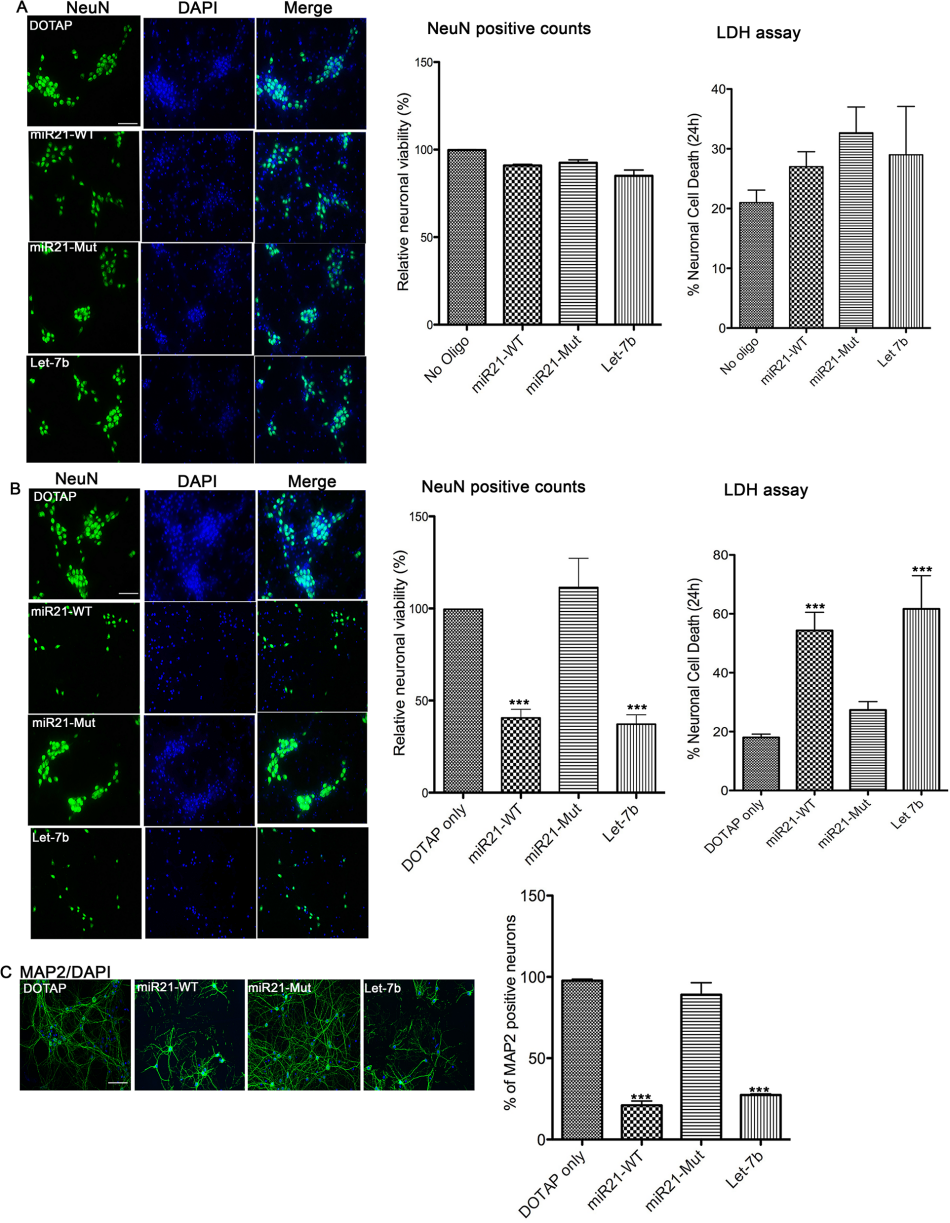
In vitro neurotoxicity assays with artificial EVs. **(A)** Wildtype (WT) mouse hippocampal neurons were incubated with 1 μg of synthetic miRNAs; miR-21 (miR21-WT), miR-21 containing a mutation in TLR7 binding site (miR21-Mut) and a known TLR7 activator, Let-7b, and DOTAP artificial EVs. Neurons were incubated for 24 hr and then stained with NeuN, a cell body marker for neurons (Left). The number of NeuN positive neurons was counted and the relative neuronal viability to untreated cultures was calculated (Middle). The result indicates no significant cell death by naked synthetic miRNAs. LDH assay was performed to assess the neuronal viability (Right). Results indicate no difference in cell death. **(B)** Synthetic miRNAs were mixed with DOTAP liposomal formulations creating “artificial EVs” and WT hippocampal neurons were incubated with 1 μg of synthetic miRNAs within artificial EVs for 24 hr. NeuN staining was performed, and the results indicated increased neuronal loss as seen in fewer numbers of green NeuN positive neurons in miR21-WT and Let-7b treated cultures when compared to miR21-mut and DOTAP treated hippocampal cultures (Left). Quantification (middle bar graph) shows a significant cell death in cultures treated with miR-21-WT and Let-7b when compared to DOTAP control. LDH assay was performed to assess the neuronal viability. Results indicate a significantly higher cell death with miR-21-WT and Let-7b than with miR-21-Mut and DOTAP control. Statistical analyses were performed on data from six independent experiments for NeuN counting and three independent experiments for LDH assay. Error bar = SEM; ^***^ *P*<0.001; One-way ANOVA with Dunnett’s post-hoc test. **(C)** Immunostaining was performed for the neuronal (neurite) marker, MAP2 and staining reveals loss of neurites in cultures treated with miR-21-WT and Let-7b artificial EVs compared to DOTAP only or in the miR-21-Mut treated cultures. Statistical analyses were performed on data from three independent experiments. Error bar = SEM; ^***^ *P*<0.001; One-way ANOVA with Dunnett’s post-hoc test. Scale bar = 20 μm.

Recent studies have found that certain microRNAs containing a GU-rich sequence could activate TLR7. Neurotoxicity and neuronal and non-neuronal cell activation has been found with such free microRNAs and with synthetic EVs of lipid-encapsulated microRNAs [25–27]. First, we asked if the presence of extracellular miR-21 could render neurotoxicity. To do so, we used miRNA oligonucleotides (oligos) of wildtype miR-21 (miR-21-WT), a mutant miR-21 (miR-21-Mut) containing a point mutation in one of the uridine residues in a small G/U sequence in the TLR binding motif (U to G, since uridines are more crucial ligands to TLRs [29]). Another characterized microRNA, the TLR7 ligand let-7b, was used as a positive control. First, we added the free “naked” oligos directly to the hippocampal neuronal cultures. Results indicated no significant cell death observed either in miR-21-WT, miR-21-Mut, or let-7b, assessed with NeuN counting or LDH assay (Fig 3A, middle and right). Next, we tested whether these microRNAs, when encased in EV-like vesicles, could have an effect on neurons. Interestingly, when the neuronal cultures were treated with these synthetic EVs, significant neuronal cell death was observed with miR-21-WT and let-7b but not with miR-21-Mut, again demonstrated by both NeuN cell counting assay and LDH assay (Fig 3B). Staining with the neuronal marker MAP2 also revealed a loss in neurites (Fig 3C). In clear distinction to what we saw with free miR-21, the delivery of miR-21 in EV-like vesicles is essential to elicit neurotoxicity.

### miR-21 acts via TLR7 to exert neurotoxicity

**Fig 4:**
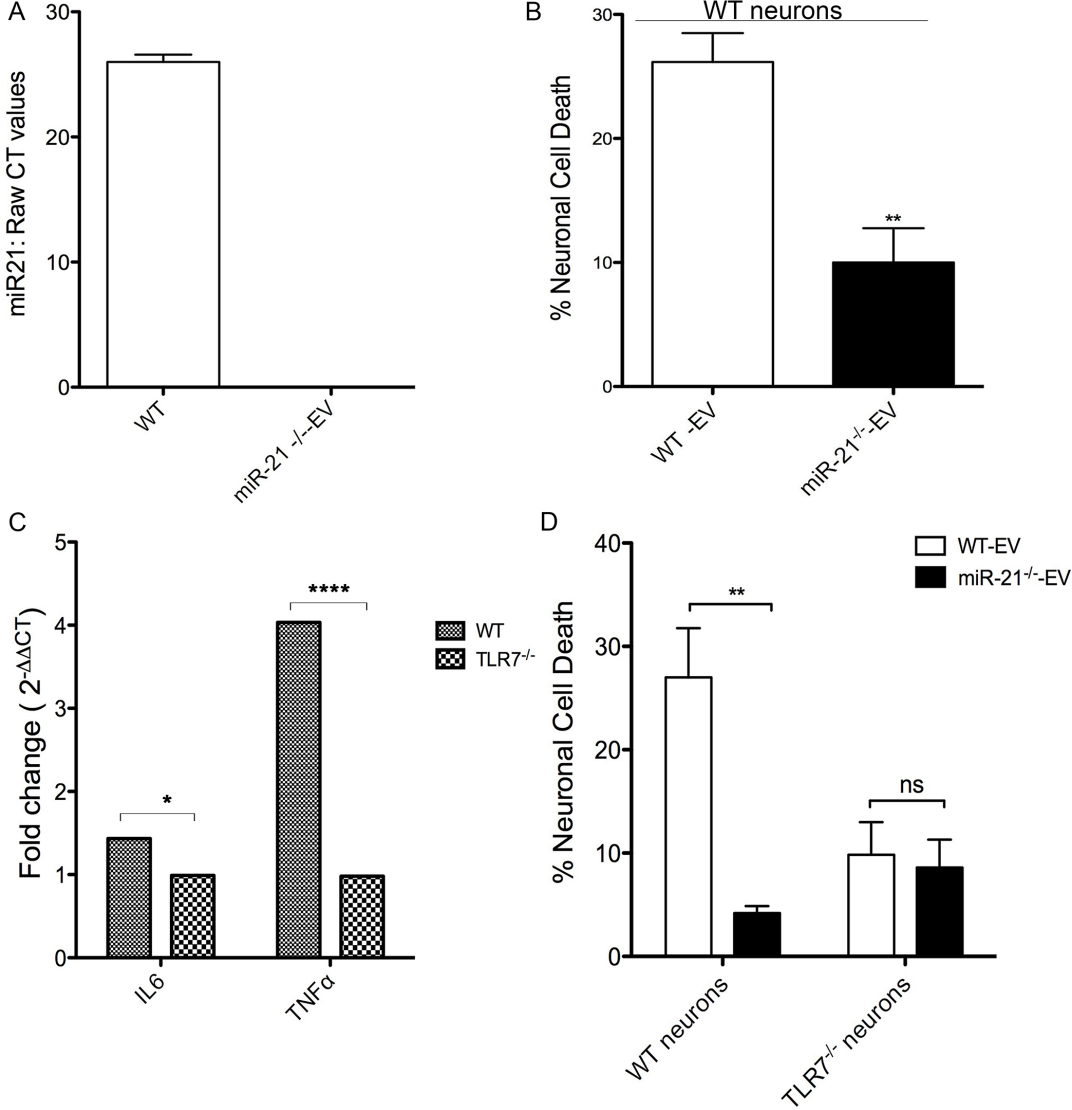
In vitro neurotoxicity assays with exosomes from bone marrow derived macrophage (BMDM) cultures. **(A)** Quantitative reat-time PCR (qRT-PCR) for miR-21 was performed on EVs isolated from WT and miR-21^-/-^ BMDMs. Raw CT values confirm the absence of miR-21 in the EVs isolated from miR-21^-/-^ BMDMs. **(B)** WT mouse hippocampal neurons were incubated with 1 μg of EVs isolated from WT (WT-Exo) and miR-21 ^-/-^ (miR-21KO-Exo) littermate BMDMs for 24 hr. LDH assay was performed to assess the neuronal viability Results indicate a significantly higher in cell death with WT EVs than with miR-21^-/-^ EVs. Statistical analyses were performed on data from six independent experiments. Error bars = SEM; ^**^ *P* < 0.01; unpaired t-test. **(C)** Cultured hippocampal neurons (DIV 7) from WT and TLR7-/- mice were treated with CL075 (6μM) and vehicle for 6h and harvested for real time PCR using GAPDH as an internal control to quantify the levels of IL6 and TNFα. Error bars = SEM; ^*^ *P* < 0.05; ****P < 0.0001; Two-way ANOVA with Bonferroni post-hoc test. **(D)** LDH assay was performed on WT and TLR7^-/-^ mice hippocampal cultures with WT and miR-21^-/-^ littermate BMDM derived EVs. A significant increase in neuronal cell death is seen with WT-EVs when compared to miR-21^-/-^ EVs. No miR-21-EV induced toxicity was found when hippocampal neurons from TLR7^-/-^ mice were used. Error bars = SEM; ^**^ *P* < 0.01; Two-way ANOVA with Bonferroni post-hoc test.

To further examine whether EV-miR-21 activates the TLR7 pathway, we isolated EVs from bone marrow derived macrophage cultures prepared from wildtype (WT) and miR-21^-/-^ mice and used these, differing in the presence of miR-21, to examine potential neurotoxicity (Fig 4A). Indeed, there is a significant increase in neuronal cell death when cultures were treated with EVs derived from WT than from miR-21^-/-^ macrophage cultures (Fig 4B). In order to examine if this neurotoxicity is dependent on TLR7, we performed the neurotoxicity studies on neurons derived from TLR7^-/-^ animals. To confirm that TLR7 ^-/-^ neurons do not respond to ligands, we treated the hippocampal neurons isolated from WT and TLR7 ^-/-^ mice with TLR7 agonist CL075. Quantitative RT-PCR on confirms the expression of pro-inflammatory cytokine genes such as *IL6* and *TNFα* only in WT neurons confirming that TLR7 ^-/-^ neurons did not respond to TLR7 ligand stimulation (Fig 4C). Treating the TLR7 ^-/-^ neurons with WT-EVs and miR-21^-/-^ EVs demonstrated that toxicity depended not only on the presence of miR-21 in the EVs but also upon the presence of TLR7 in the neurons (Fig 4D). These results clearly indicate that both miR-21 and TLR7 are required for the activation of neurotoxic pathways.

### EVs isolated from SIVE brains cause neurotoxicity and can activate TLR7 signaling pathway

**Fig 5:**
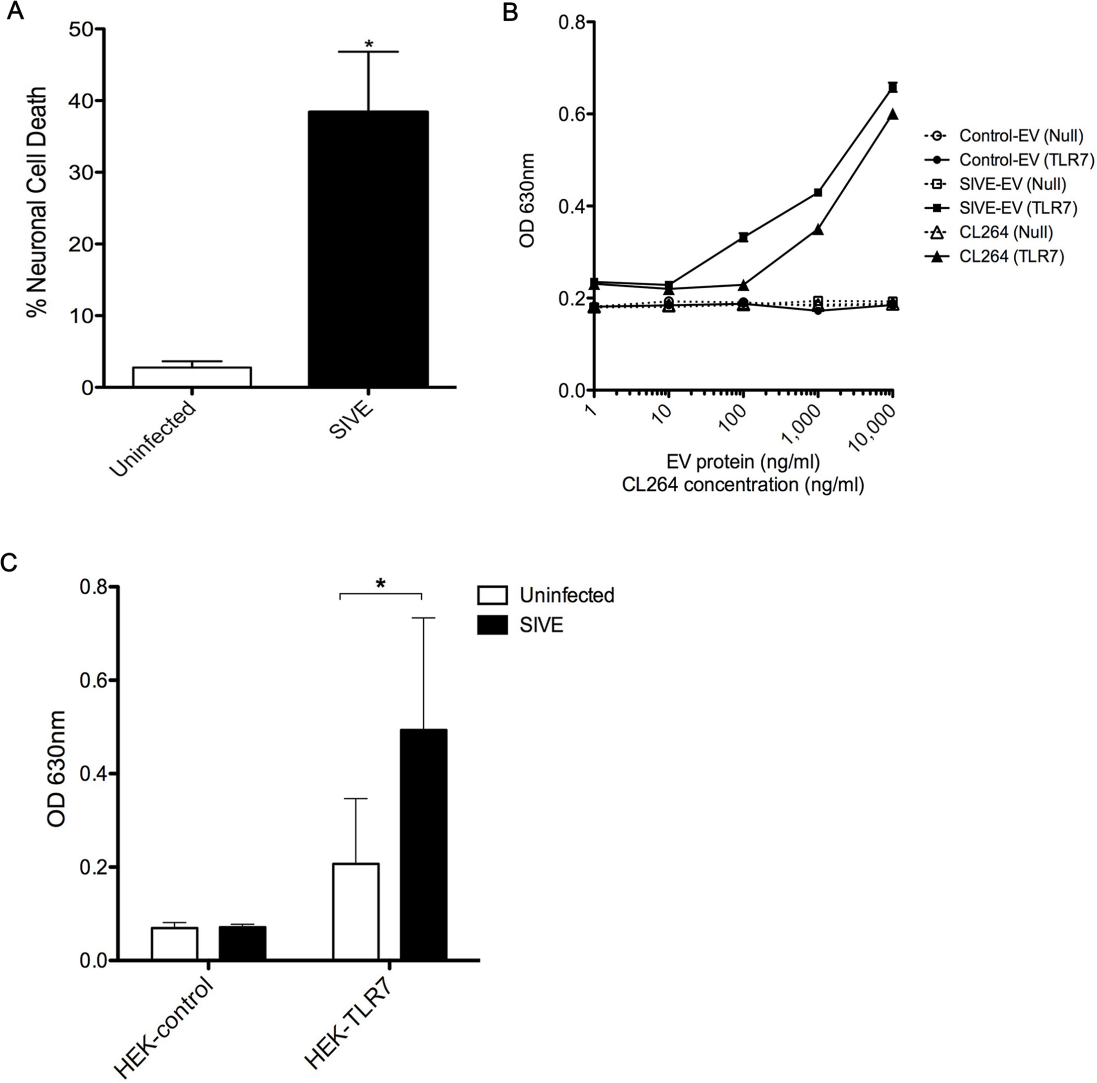
EVs from SIVE brains can elicit neurotoxicity and can activate TLR7 pathway. **(A)** Wildtype (WT) mouse hippocampal neurons were incubated with 1 μg of EVs isolated from uninfected and SIVE brains for 24 hr. LDH assay was performed to assess the neuronal viability; results indicate a significant increase in cell death with SIVE than with uninfected EVs. Statistical analyses were performed on data from three independent sets. Error bars = SEM; ^*^ *P* < 0.05; unpaired t-test. **(B)** HEK-Blue Null (HEK-control) or TLR7 overexpressing (HEK-TLR7) cells were incubated with increasing concentrations of EVs from uninfected and SIVE brains and with CL264. Dose response curves show a clear increase in the SEAP activity with SIVE EVs in TLR7 (SIVE-EV (TLR7)), comparable to the TLR7 ligand, CL264. No response was seen with EVs from control monkey brains (Control-EV (TLR7)) cells nor in any of the conditions using the cells without TLR7 expression (Null). **(C)** Similar to (B), cells were incubated with 1 μg of EVs isolated from uninfected and SIVE brains for 24 hr. Increased activity was found in HEK-TLR7 cells treated with SIVE EVs when compared to uninfected EVs. No change in absorbance was observed in HEK-Null (Control) cells. Statistical analyses were performed on data from three independent experiments. Error bars = SEM; ^*^ *P* < 0.05, Two-way ANOVA with Sidak’s multiple comparison was performed.

Since miR-21 is increased in EVs from the brains of monkeys with SIVE, and EV associated miR-21 (EV-miR-21) is associated with neurotoxicity, we then assessed whether EVs isolated from the SIVE (SIVE-EV) and uninfected (control-EV) brains would show differences in neurotoxicity. Indeed, treatment of neuronal cultures with SIVE-EV significantly increased neuronal death as compared to control-EV (Fig 5A).

Next, we asked if the TLR7 pathway is activated by EV-miR-21. Using HEK (human embryonic kidney) cell lines that expressed, or not, TLR7, in addition to a reporter gene (secreted alkaline phosphatase), we first examined the signaling of the EVs derived from SIVE brains (as well as use of CL264, a TLR7 agonist). The results indicated a dose dependent signaling with TLR7, which was not seen with EVs from uninfected brains (Fig 5B and 5C).

### Necrostatin-1, a necroptosis inhibitor, prevents neuronal cell death caused by miR-21

**Fig 6:**
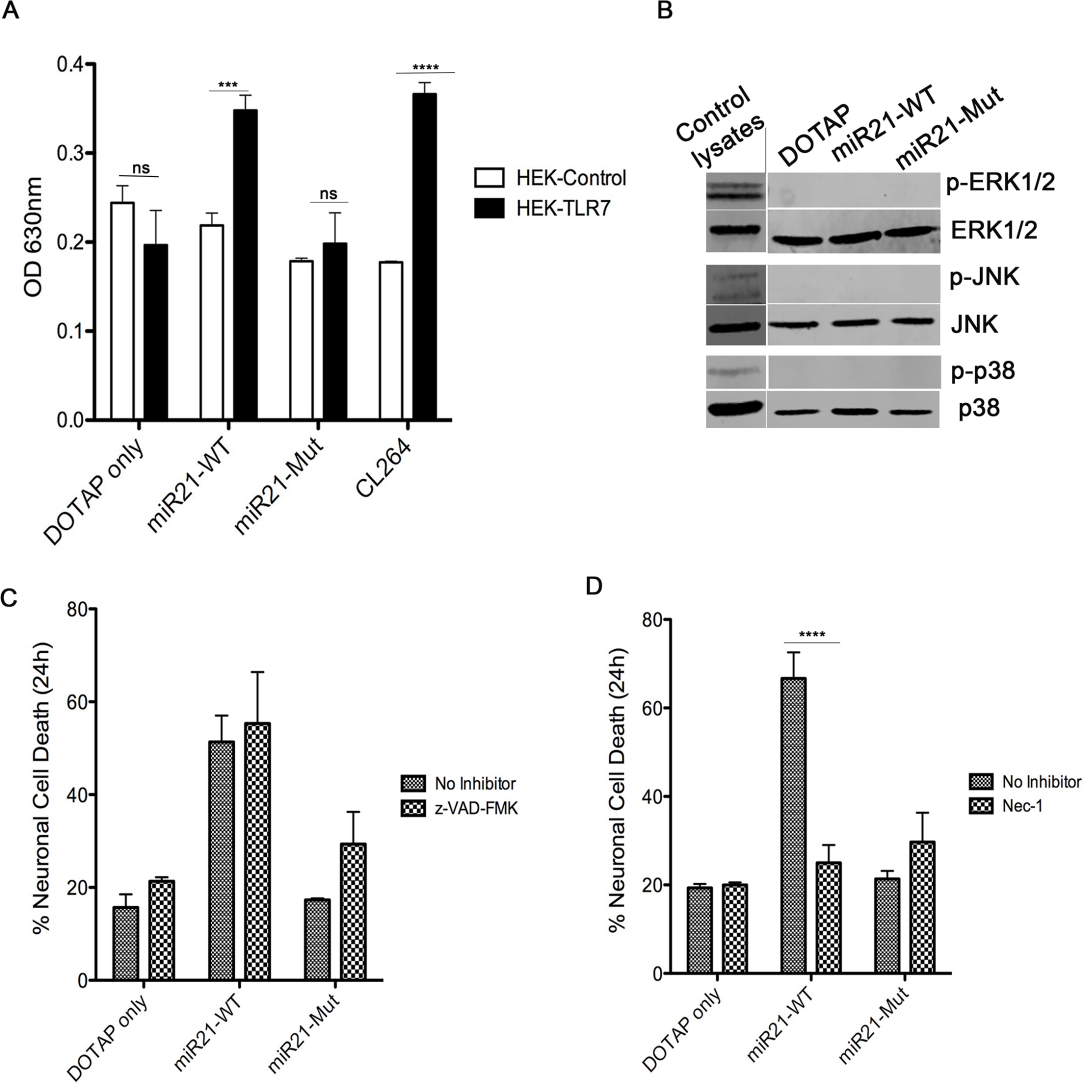
miR-21 neurotoxicity is rescued by Nec-1, a necroptosis inhibitor. **(A)** HEK-Blue Null (HEK-control) or TLR7 overexpressing (HEK-TLR7) cells were incubated with 1 μg of synthetic miRNAs; miR21-WT, miR21-Mut, and CL264, a TLR7 ligand. Neurons were incubated for 24 hr followed by measurement of the secreted alkaline phosphatase (SEAP) enzyme activity (measured as absorbance at 630nm) as a read out of TLR7 activation. The results indicate a significant increase when TLR7 expressing cells were treated with miR21-WT, Let-7b and CL264; whereas, no difference was seen in DOTAP control and miR21-Mut. Statistical analyses were performed on data from three independent experiments. Error bars = SEM; *** *P* <0.001, **** *P* < 0.0001; two-way ANOVA with Bonferroni post-hoc test. **(B)** Wildtype (WT) mouse hippocampal neurons were incubated with 1 μg of synthetic miRNAs; miR-21 (miR21-WT) and miR-21 containing a mutation in TLR7 binding site (miR21-Mut) and DOTAP artificial EVs for 24 hr. Neurons were harvested and lysates were loaded for Western blotting to look at the activation of MAPK signaling proteins, p-ERK1/2, p-JNK and p-38. As the results indicate, no difference in expression of proteins was seen. Positive control lysates were used to check the specificity of the antibodies. **(C)** WT mouse hippocampal neurons were pre-treated for 1 hr with 10 μM z-VAD-fmk. After pre-treatment, neurons were treated simultaneously with 1 μg of synthetic miRNAs; miR-21 (miR21-WT), miR-21 containing a mutation in TLR7 binding site (miR21-Mut) and DOTAP artificial EVs. LDH assay indicate that z-VAD-fmk could not rescue the neurons from miR-21 induced cell death. **(D)** Similar to (C), neurons were pretreated for 1 hr with necroptosis inhibitor, Necrostatin-1 (Nec-1) and then incubated simultaneously with 1 μg of synthetic miRNAs; miR-21 (miR21-WT), miR-21 containing a mutation in TLR7 binding site (miR21-Mut) and DOTAP artificial EVs different artificial EVs. Results indicate that Nec-1 was able to protect neurons from undergoing cell death by miR-21 containing artificial EVs. Error bars = SEM; **** *P* < 0.0001; two-way ANOVA with Bonferroni post-hoc test.

In order to determine if the miR-21 induced TLR7 signaling, HEK-TLR7 cells were treated with EV-like vesicles. Results indicate that miR-21 induced signaling but not the vehicle control or the miR-21 mutant (miR-21-Mut) (Fig 6A). We next examined if the cell death observed in the EV-miR-21 treated neuronal cultures occurs via apoptosis. Since the trigger of apoptosis involves activation of the mitogen activated protein kinase (MAPK) signaling pathway, that transduces signals to the nuclear transcription factor NF-κB, we first looked at the expression of these proteins. Western blot analysis revealed that none of the signaling proteins such as p-ERK1/2, p-JNK and p-p38 changed by treatment with miR-21 WT EVs (Fig 6B). Next, we treated the hippocampal neurons with a pan-caspase inhibitor, z-VAD-fmk, which have been shown previously to the neurotoxicity resulting from let-7b treatment [25]. However treatment of hippocampal neuronal cultures with z-VAD-fmk did not prevent neuronal cell death (Fig 6C). A caspase-independent form of programmed cell death, termed necroptosis, has been recently identified to play a role in disorders of the central nervous system and elsewhere [30]. Necroptosis occurs through a signaling cascade dependent upon receptor interacting protein kinase-1 (RIPK-1). To determine if necroptosis was involved in the neurotoxicity induced by miR-21, we treated the cultures with necrostatin-1, which specifically inhibits RIPK-1. Indeed the LDH assay results indicate that Nec-1 was able to prevent EV-miR-21 induced neurotoxicity in hippocampal neurons (Fig 6D). Hence the necroptotic, rather than apoptotic, pathway is active in EV-miR-21 induced neurotoxicity.

## Discussion

In this present study, we showed that EV-miR-21 could activate the TLR7 signaling pathway thus leading to neurotoxicity in SIV neuropathogenesis. Through RNA sequencing on EVs isolated from control and SIVE brains, we found differences in several miRNAs, the most striking being miR-21. Previously, we showed that miR-21 is significantly increased in neurons. Here, we significantly expand this to reveal the presence of miR-21 in brain EVs from macaques with SIV neuropathogenesis. In the diseased brain, microglial/macrophages express miR-21; and in vitro, macrophage produces EVs containing miR-21. We found that miR-21 when associated with EVs exhibit neurotoxicity, and this neurotoxicity is dependent upon neuronal expression of TLR7. Furthermore, we also discovered that neurotoxicity by EV-miR-21 is not caused by an apoptotic mechanism but through the activation of a programmed necrotic pathway termed necroptosis.

Brain macrophages are the most likely source for EV-miR-21, although we cannot exclude the possibility that neurons to secrete miR-21 associated EVs as well. Several lines of evidence suggest that miR-21 is upregulated during inflammation in the brain [24,31,32]. For the first time, we report that miR-21 is upregulated in EVs in the diseased brain and can activate TLR signaling in neurons during SIV infection.

TLR7, similar to other TLRs, is a pattern recognition receptor, and plays a role in pathogen recognition as part of the innate immune system. TLR7 is endosomally located and recognizes single stranded RNA (ssRNA) in mice and humans; TLR8 also recognizes ssRNA in humans. TLR7 and TLR8 are related phylogenetically and functionally and have been identified as important sensors of ssRNA from the viral genomes of influenza and vesicular stomatitis virus as well as HIV itself [29,33,34]. These sequences can specifically activate TLR7 in mice and TLR7/TLR8 in humans [33]. A number of studies have revealed that several miRNAs, such as miR-21, miR-29a and let-7b, can even serve as physiological ligands of the ssRNA-sensing [25–27]. Ours is the first study so far that has tested this possibility in the context of SIV infection in the brain.

Through the repression of its targets, miR-21 was shown previously to act as both pro-apoptotic [35] and anti-apoptotic miRNA [36]. Previously, we showed that miR-21 causes alterations in neuronal physiology by acting through its target gene MEF2C [11]. Expanding upon its pathogenic actions, in this study we found that miR-21 is released via EVs and that it can directly activate neurotoxic signaling pathways by activating TLR7 receptors in the neuron. Using in vitro constructed EVs, EVs from mouse macrophages, and EVs isolated from primate brains, we provide multiple lines of evidence revealing EV-miR-21 signaling through TLR7, resulting in neuronal demise.

Previously, it was shown that “naked” let-7b synthetic oligonucleotide elicited neurotoxicity [25,27]. However, in our cultures, we could not see significant neurotoxicity by naked let-7b (Fig 3A). Enclosing let-7b in DOTAP as an EV-like particle, however, resulted in neurotoxicity. A recent study on the role of let-7b in activation of nociceptor dorsal root ganglion (DRG) neurons indicated that cell surface expression of TLR7 and another receptor (TRPV) were necessary for the effect [27]. Hence, the localization of the TLR7 in the cells, and its interaction with other receptors, might be important for miRNA-mediated activation of signaling pathways such as neurotoxicity and the potential actions of free versus EV-miRNA.

Additionally, several other factors present in EVs were shown to mediate inflammatory responses and neurotoxic pathways, and EVs may contain proinflammatory mediators that could contribute to pathogenesis and progression of HAND [37–39]. In neurodegenerative diseases such as Prion disease, Parkinson’s and Alzheimer’s, toxic factors such as prions, tau, amyloid β, α-synucleins, aggregates of superoxide dismutase 1 were shown to be present in EVs eliciting neurotoxicity [40]. It is also unclear as to why miR-21 is localized to specific cell types in the brain, either through its production or its uptake from EVs. Intriguingly, temporal differences in expression patterns have been detected in neurons and astrocytes after ischemic injury, where the miR-21 increase in neurons was much later when compared to astrocytes, which occurred 12 hr post injury [41]. Given the more chronic nature of SIV infection, such temporal differences in the response could not be detected in our experiments.

To study pathways potentially activated upon treatment with EV-miR-21 leading to neurotoxicity, we first looked at changes in the phosphorylation of signaling proteins such as ERK, JNK and p-38 in the MAPK pathway. The MAPKs are a family of kinases that transduce signals from the cell membrane to the nucleus in response to a wide range of stimuli, including stress (reviewed in [42]). Interestingly, we did not find any significant changes in the protein expression of signaling proteins belonging to the MAPK pathway. MAPK activation is linked to apoptosis accompanied by caspase activation, in parallel with not finding activation of MAPK members treatment with a pan caspase inhibitor, z-VAD-fmk, did not rescue the neurons from undergoing death indicating that EV-miR-21 caused neurotoxicity by activating a different cell death pathway. Intriguingly, a novel cell death pathway has been reported recently that causes cell death by a regulated necrosis, termed necroptosis [43–46]. Death receptors [47], interferons, toll-like receptors (TLRs) [48], intracellular RNA and DNA sensors [49], and probably other mediators induce this pathway. Necroptosis is a programmed necrosis that requires a number of regulatory proteins and a key protein, RIPK1. RIPK1 has important kinase-dependent and scaffolding functions that inhibit or trigger necroptosis and apoptosis. The development of the RIPK1 inhibitor Nec-1 has been a major breakthrough in research on necroptosis, and the first disease model in which the role of necroptosis was investigated was ischemic brain injury [50]. Studies in several other disease models revealed that Nec-1 was able to prevent cell death in cells undergoing necroptosis [30]. Hence, we tested to see if Nec-1 will be able to rescue neuronal death triggered by EV-miR-21. Indeed we observed that pretreatment with Nec-1 was able to prevent neurons from undergoing death. Hence for the first time we report that a miRNA (miR-21) in EVs could cause cell death through a necroptotic cell death pathway. Further studies need to be conducted to ascertain the pathway components activated or involved in initiating necroptosis, and whether necroptosis inhibitors may be useful in vivo to lead to clinical studies.

In the era of combination antiretroviral therapy, HAND continues to be a common morbidity among individuals infected with HIV. While the severity of the disease has decreased dramatically, it is still poorly understood as to why the milder forms of HAND are prevalent in HIV-1 infected individuals. The inflammatory condition in the brain due to the continued viral presence is one possible explanation for CNS damage [51]. It is interesting that a significant change in miR-21 levels was not seen in animals without CNS disease, which is in support with studies referring to miR-21 as a critical player in inflammation. It was shown previously that miR-21 levels markedly increased during tissue injury and inflammation in the heart [52], spinal cord [53], neurons and astrocytes [41], and in traumatic brain injury [54–58]. Furthermore, it has been already shown that pro-inflammatory cytokine signaling, such as IL6 via the activation of STAT3 promoter, increases miR-21 [59]. In SIVE brains, there is a marked inflammatory cytokine response to the presence of the virus; and therefore, up regulation in miR-21 levels could be expected.

In summary, our study for the first time provides evidence of differences in EV derived miRNAs in CNS disease. We found increased miR-21 expression in EVs derived from SIVE brains when compared to controls. We also report for the first time that EV-miR-21 causes neurotoxicity by activating necroptosis, a novel cell death pathway. The studies presented here are novel findings in neuroAIDS research, and the results implicate EVs as crucial communicators between various cells in the brain. In the context of HIV infection, they are mediators of many neurotoxic factors, miR-21, being one of them. This study will further form a premise for therapeutic studies for prevention of long-term neuronal damage as seen in HAND.

## Materials and Methods

### Ethics statement

Materials used in these studies were from animal work performed under Institutional Animal Care and Use Committee approval (Protocol #: 08-034-07-FC and 11-032-05-FC) from the University of Nebraska Medical Center. Animal welfare was maintained by following the National Institutes of Health Guide for the Care and Use of Laboratory Animals (National Research Council of the US National Academy of Sciences) and US Department of Agriculture policies by trained veterinary staff and researchers under Association for Assessment and Accreditation of Laboratory Animal Care certification, insuring standards for housing, health care, nutrition, environmental enrichment and psychological well-being. Primary enclosures consisted of stainless steel primate caging provided by a commercial vendor. Animal body weights and cage dimensions were regularly monitored. Overall dimensions of primary enclosures (floor area and height) met the specifications of The Guide for the Care and Use of Laboratory Animals, and the Animal Welfare Regulations (AWR’s). Light cycle was controlled at 12/12 hours daily. All animals were fed standard monkey chow diet supplemented daily with fruit and vegetables and water ad libitum. Social enrichment was delivered and overseen by veterinary staff and overall animal health was monitored daily. Animals showing significant signs of weight loss, disease or distress were evaluated clinically and then provided dietary supplementation, analgesics and/or therapeutics as necessary. These met or exceeded those set forth in the Guide for the Care and Use of Laboratory Animals from the National Research Council of the US National Academy of Sciences. Archived tissue used in these studies was from animal (Macaca mulatta) studies performed under Institutional Animal Care and Use Committee approval from the University of Nebraska Medical Center. Animal welfare was maintained by following the National Institutes of Health Guide for the Care and Use of Laboratory Animals. All efforts were made to ameliorate suffering of the animals, including the use of anesthesia with ketamine, xylazine and phenobarbital at necropsy.

### Reagents

The following oligoribonucleotides were synthesized by Integrated DNA Technologies (Coralville, IA, USA) using all phosphorothioate linkages to protect from degradation, and methyl groups on the 5’ and 3’ nucleotides. The changed base in miR21-mut (U to G at position 20) is underlined. All were used following HPLC purification.

miR21-WT: 5'- UAG CUU AUC AGA CUG AUG UUG A -3'; miR21-Mut: 5'- UAG CUU AUC AGA CUG AUG UGG A -3'; and let-7b, 5’- UGA GGU AGU AGG UUG UGU GGU U -3′. Necrotstatin-1, a necroptosis inhibitor and z-VAD-fmk, pan-caspase inhibitor, were purchased from Enzo lifesciences (Farmingdale, NY, USA).

### Mice and cell lines

miR-21^−/−^ and *Tlr7* ^−/−^ mice were purchased from Jackson Laboratories (Bar Harbor, Maine) and bred in the UNMC animal facility. Pregnant WT mice were purchased from Charles River (Wilmington, MA, USA). HEK-Blue TLR7 cells designed for studying the stimulation of TLR7 by monitoring the activation of NF-κB and AP-1 were cultured in DMEM supplemented with 10% FBS, normocin (50 μg/ml), blasticidin (10 μg/ml), zeocin (100 μg/ml) (InvivoGen, San Diego, CA). Cells were grown at 37° C in humidified air with 5% CO_2_. Control HEK-Blue Null cells were cultured similarly except without zeocin.

### SIV/rhesus monkey model

Samples from SIV-infected rhesus monkeys that developed SIVE, and from uninfected control monkeys, were obtained from previous studies. For animals used in this study, the infection was allowed to follow its natural course, and animals were euthanized when they showed signs of simian AIDS. At necropsy, all animals were perfused with PBS containing 1 U/ml heparin to remove blood-borne cells from the brain, and samples were taken and stored at -80° C. Those in which pathological examination revealed multinucleated giant cells, microglial nodules and infiltration of macrophages into the brain were classified as having SIVE. Samples from these animals, as well as uninfected control animals were prepared in a similar manner, were used for this study.

### Fluorescent *in situ* hybridization (FISH) and immunofluorescent (IF) labeling

FISH and IF were performed as described previously [60]. First, formalin-fixed paraffin-embedded sections were deparaffinized. For combined FISH and IF, this was followed by antigen retrieval using 0.01 M citrate buffer and postfixation using 0.16 M l-ethyl-3-(3-dimethylaminopropyl) carbodiimide (EDC; Sigma-Aldrich, St. Louis, MO, USA) to prevent loss of small RNAs. The sections were incubated with hybridization buffer (50% formamide; 10 mM Tris-HCl, pH 8.0; 200 μg/ml yeast tRNA; 1× Denhardt's solution; 600 mM NaCl; 0.25% SDS; 1 mM EDTA; and 10% dextran sulfate) for 1 hr at 37° C in a humidified chamber for prehybridization. They were then incubated overnight at 37° C with locked nucleic acid (LNA)-modified DNA probes, all labeled with digoxigenin at the 5′- and 3′-termini (Exiqon, Woburn, MA, USA). Probes were used at a concentration of 4 pmol of probe per 100 μl of hybridization buffer. The sequences of the probes are; U6: CAC GAA TTT GCG TGT CAT CCT Y; miR-21: 5’- TCA ACA TCA GTC TGA TAA GCT A -3’; Scramble (Scr) 5’- GTG TAA CAC GTC TAT ACG CCC A -3’. Stringency washes were performed with 2× and 0.2× SSC (Invitrogen, Carlsbad, CA, USA) at 42° C. The hybridization and wash temperatures were optimized in preliminary experiments. The sections were then blocked with a solution of 1% BSA, 3% normal goat serum in 1× PBS for 1 hr at room temperature, followed by incubation with anti-digoxigenin peroxidase antibody (1:100 in blocking buffer; Roche Applied Science, Mannheim, Germany) overnight at 4° C. For combined FISH and IF, co-incubation with either anti-CD163 (1:100; Vector Labs, Burlingame, CA, USA) or anti-glial fibrillary acidic protein (GFAP; 1:2000; Dako, Glostrup, Denmark) was performed at this step. The following secondary antibodies were used: 568 donkey anti-rabbit and 488 goat anti-mouse IgG (1:400; Invitrogen). This was followed by signal amplification using tyramide signal amplification Cy5 kit (Perkin Elmer, Waltham, MA, USA) according to the manufacturer's protocol. The slides were mounted in Prolong gold antifade reagent with DAPI (Invitrogen). The sections were imaged in Zeiss Observer.Z1 microscope equipped with a monochromatic Axiocam MRm camera using Axiovision 40 v.4.8.0.0 software (Carl Zeiss, Oberkochen, Germany). The following colors were assigned to the fluorescent signals using the Axiovision software: Green for CD163, Red for GFAP, Magenta for Cy5, Blue for DAPI.

### Extracellular vesicle (EV) isolations

EV isolations from the brains were carried out as described previously with modifications [28]. Previously, dissected and frozen macaque brain tissues (weighing approximately 500 mg each) were dissected and treated with 20 units/ml papain (Worthington, Lakewood, NJ) in Hibernate A solution (5 ml/hemi-brain; BrainBits, Springfield, IL, USA) and rocked for 15 min at 37° C. The brain tissue was gently homogenized in 10 ml/brain of cold Hibernate A solution. The brain homogenate was sequentially filtered through a 40 μm mesh filter (BD Biosciences, San Jose, CA), a 5 μm filter (Pall Corporation, Port Washington, NY) and a 0.2 μm syringe filter (Thermo Scientific, Waltham, MA). EVs were isolated from the filtrate as described previously [15,28]. Briefly, the filtrate was sequentially centrifuged at 300 × *g* for 10 min at 4° C, 2000 × *g* for 10 min at 4° C, and 10,000 × *g* for 30 min at 4° C to discard cells, membranes and debris. The supernatant was centrifuged at 100,000 × *g* for 60 min at 4° C to pellet EVs. The EV pellet was resuspended in 37 ml of cold PBS (Thermo Scientific, Waltham, MA), and the EV solution was centrifuged at 100,000 × *g* for 60 min at 4° C. The washed EV pellet was resuspended in 2 mL of 0.95 M sucrose solution and inserted inside a sucrose step gradient column (six 2 ml steps starting from 2.0 M sucrose down to 0.25 M sucrose in 0.35 M increments, with the 0.95 M sucrose step containing the EVs). The sucrose step gradient was centrifuged at 200,000 × *g* for 16 hr at 4° C. A 1 ml fraction was collected from the top of the gradient and discarded, and 6 mL of the gradient were collected in the EV rich layers containing material with density higher than 1.07 (0.60 M sucrose layer) and lower than 1.17 (1.30 M sucrose layer) [28]. Pooled fractions were diluted to 30 ml with cold PBS. 25 ml of this volume was taken for RNA extraction, and 5 ml used for Western blot studies. Sample volumes were brought up to appropriate volumes with cold PBS and centrifuged at 100,000 × *g* at 4° C for 60 min. PBS was pipetted off both pellets. Protein pellet was suspended in 50 to 100 μl of PBS depending on pellet size.

EV isolations from BMDM preparations were carried out by Exoquick (SBI) according to manufacturer’s instructions.

### Electron microscopy

For transmission electron microscope (TEM), a 10 μl drop of EV sample was placed on the grid (200 mesh copper grids coated with Formvar and silicon monoxide) and allowed to sit for 2 min. The excess solution was drawn off by filter paper, and the remaining thin film of sample was allowed to dry for 2 min. A drop of NanoVan negative stain was placed on the grid for 1 min. The excess negative stain was then drawn off by filter paper and allowed to dry for at least 1 min before being placed in the TEM. Grids were examined on a Tecnai G2 Transmission Electron Microscope (built by FEI, Hillsboro, Oregon, USA) operated at 80Kv.

### RNA sequencing

Small RNAseq was performed by LC Sciences (Houston, TX, USA). Using the RNA isolated from EVs, a small RNA library was generated using the Illumina Truseq Small RNA Preparation kit following the manufacturer’s guidelines. The cDNA library was purified and used for cluster generation on Illumina’s Cluster Station and then sequenced on the Illumina GAIIx (Ilumina, San Diego, CA). Raw sequencing reads were obtained using Illumina’s Sequencing Control Studio software (version 2.8) following real-time sequencing image analyses and base-calling by Illumina's Real-Time Analysis (version 1.8.70). A pipeline script, ACGT101-miR v4.2 (LC Sciences), was used for sequencing data analyses [61–63]. Sequences were then mapped to miRbase (version 20.0) [64]. 636 unique sequences mapped to both *Macaca mullata* mirs in miRbase and the *Macaca mullata* genome. Many of these had very low normalized counts (median/mean for Control, SIV, and SIVE were 6.34/444.28, 5.17/435.8, and 6.04/554.50 respectively); thus, only those with >500 counts in any one group (comprising 75 mirs) were chosen for statistical analyses. To assess differences between the groups, normalized sequence counts were subjected to a Bayes-regularized one-way ANOVA using analysis conducted using the Cyber-T web server (http://cybert.ics.uci.edu) [65,66]. The sliding window size was set at 101, the Bayesian confidence value was 11, and analysis was performed on the natural logarithm of the values. Significant changes were assigned if the Bonferroni corrected p value was <0.05.

### Real-time quantitative RT-PCR (qRT-PCR)

For quantification of miRNA in EVs by qRT-PCR, TaqMan mature miR assays (Applied Biosystems, Carlsbad, CA, USA) were used according to the manufacturer's protocol. The relative amount of miR-21 was determined by comparison to a standard dilution curve, made from a cDNA preparation from a miR-21 overexpressing cell line. The Ct values of the samples were extrapolated into the standard curve to calculate the relative copy number. We used the formula [RNA/DNA] = 10^Ct-b/m (where Ct = threshold Ct value, b = Y-intercept and m = slope) to calculate the amount of miRNA in each sample.

### Western blotting

Exosomal lysates were prepared using RIPA buffer (50 mM Tris/HCl, pH 8; 150 mM NaCl; 1% Nonidet P-40; 0.5% sodium deoxycholate; and 0.1% SDS), and protein quantification was carried out using Pierce BCA protein assay (Thermo Scientific, Rockford, IL, USA). Protein (5–15 μg) was loaded in each lane of NuPAGE 4–12% Bis-Tris gels (Invitrogen). For EV proteins, all the blots for tetraspanins (CD9, CD63, CD81) were run on non-reducing gels as described previously [67–69] and flotillin, HSP70 and TSG101 were run under reducing conditions. Separated proteins were transferred onto nitrocellulose membranes using iBlot (Invitrogen). The membranes were blocked in SuperBlock (TBS) blocking buffer (Thermo Scientific) and then incubated overnight at 4° C with primary antibody. The following primary antibodies were used: rabbit polyclonal anti-Flottilin (Abcam, Cambridge, MA, USA), anti-CD9 (Systems Biosciences (SBI), Mountain view, CA, USA), anti-CD63 (SBI), anti-CD81 (SBI), TSG101 (SBI), HSP70 (SBI) Synaptophysin (Thermo Scientific), SNAP-25 (Cell signaling technology (CST), Boston, MA, USA) and all the signaling antibodies (ERK1/2, pERK1/2, JNK, p-JNK, p-38, p38) and the positive control lysates were purchased from CST. This was followed by incubation with secondary antibody: HRP conjugated anti-rabbit IgG (1:20,000; Thermo Scientific) or anti-mouse IgG (1:20,000; Thermo Scientific) for 1 hr at room temperature. Blots were developed using SuperSignal West Pico Chemiluminescent Substrate (Thermo Scientific), imaged and quantified using Carestream MI software (Carestream Health Inc, Rochester, NY, USA).

### Hippocampal neuronal isolation

Primary hippocampal cultures were isolated from P0/P1 mice as described previously [70]. In brief, hippocampi were dissected and washed 3x with ice-cold calcium–magnesium-free Hanks' balanced salt solution followed by incubation with 0.25% trypsin for 20 min in a 37° C water bath. Followed by subsequent washes with HBSS and complete neuronal media (neurobasal medium containing 0.5 mM l-glutamine and B27 supplement (Life Technologies, Grand Island, NY)). Individual cells were mechanically isolated by trituration in complete neuronal media with a fire-polished glass pipette. The cells were plated on poly-d-lysine-coated coverslips/plates and cultured in at 37° C in a humidified atmosphere of 5% CO_2_ incubator.

### In vitro neurotoxicity

Neurotoxicity assays were performed as described previously with modifications [25]. Synthetic RNAs were diluted in HBS buffer (20 mM HEPES, 150 mM NaCl, pH 7.4) or encased in artificial EVs using *N*-[1-(2,3-Dioleoyloxy)propyl]-*N*,*N*,*N*trimethylammonium methylsulfate (DOTAP) (1811177; Roche, Basel, Switzerland). DOTAP was first diluted in HBS- for 5 min before mixing with an equal volume of HBS containing the RNA. The resulting mix was incubated for 20 min and 50 μl were added per well of a 24-well plate, resulting in a final volume of 200 μl. Transfections were conducted in triplicate in all experiments. The ratio of DOTAP to ssRNA was 3:1 (3 μl DOTAP to 1 μg RNA).

For toxicity studies, reagents were added to cell cultures for 24 hrs. For EV toxicity assay, 1 μg of EV preparations were added to 2 × 10^5^ neurons/well in a 24 well plate. Control cultures were incubated with phosphate-buffered saline. After 24 hr, lactate dehydrogenase (LDH) assay was conducted on the media according to the manufacture’s instructions (Cytotoxicity detection kit (LDH), Roche, Basel, Switzerland). Briefly, 100 μL of culture medium were transferred to a new 96 well plate. 100 μL of the reaction solution from the kit, containing the detection dye and the catalyst, were then added; absorption was measured after 30 min at 490 nm with 655 nm as reference wavelength. A positive control, 2% triton was used leading to 100% cytotoxicity by lysing the cells completely. The background values from wells without cells were subtracted and average values for the triplicates calculated. Cytotoxicity was then calculated according to the following equation: Cytotoxicity (%) = (experimental value–media control)/(positive control–media control) × 100. The cells were also immunostained with antibody to NeuN (Millipore, Billerica, MA, USA). For each condition, experiments were performed in duplicates. NeuN-positive cells were counted by analyzing five high-power fields per coverslip. The viability of control cells was set to 100%. The numbers of NeuN-positive cells observed for each condition were compared with control conditions, and results were expressed as relative neuronal viability.

For rescue experiments, the hippocampal neurons isolated from WT and TLR7^-/-^ mice were pretreated with necrostatin -1 (5 μM) and z-VAD-fmk (10 μM) or a vehicle control for 1 hr followed by treatment with DOTAP formulations. The inhibitors were stayed in the culture along with DOTAP formulations. LDH assay was performed to assess the neuronal viability.

### Secreted embryonic alkaline phosphatase (SEAP) assay

HEK Blue murineTLR7 293 cells and control HEK-Null (control) cells were seeded at the concentration of 2 × 10^5^ cells/well. 1 μg of EV preparations were added to the cells and incubated for another 24 hr. After 24 hr, detection medium (QUANTI-Blue) was added to the plate. The levels of SEAP (secreted alkaline phosphatase) produced by the activation of TLR7 quantitatively using a spectrophotometer at 630 nm.

## Supporting Information

S1 Table Statistical analysis of monkey brain exosomal miRNA. Identified miRNA with >500 counts in any one group (uninfected, SIV, SIVE) were analyzed for statistical significance using a Bayes-regularized one-way ANOVA, followed by Tukey’s HSD test. Columns indicate the microRNA name, the f-test statistic (Fstat, with 2 between and 38 within degrees of freedom), the p value associated with the f-test, the Bonferroni corrected value, and the values for the Tukey’s HSD test between the groups. Indicated in red are p values <0.05 for the ANOVA, Bonferroni correction, and the Tukey’s test for the miRNAs in which the Bonferroni corrected p value for the ANOVA is <0.05.
(XLSX)
