# Correction: MiR-21 in Extracellular Vesicles Leads to Neurotoxicity via TLR7 Signaling in SIV Neurological Disease

**DOI:** 10.1371/journal.ppat.1005131

**Published:** 2015-09-01

**Authors:** Sowmya V. Yelamanchili, Benjamin G. Lamberty, Deborah A. Rennard, Brenda M. Morsey, Colleen G. Hochfelder, Brittney M. Meays, Efrat Levy, Howard S. Fox

The e-mail address provided for the corresponding author is incorrect. The correct e-mail address is the following: syelamanchili@unmc.edu
