# Correction: MiR-21 in Extracellular Vesicles Leads to Neurotoxicity via TLR7 Signaling in SIV Neurological Disease

**DOI:** 10.1371/journal.ppat.1007068

**Published:** 2018-05-14

**Authors:** Sowmya V. Yelamanchili, Benjamin G. Lamberty, Deborah A. Rennard, Brenda M. Morsey, Colleen G. Hochfelder, Brittney M. Meays, Efrat Levy, Howard S. Fox

The authors elected to make all the RNA sequence data available via a repository. As a result, the data availability statement is incomplete. The full new data availability statement should read: The RNA sequence data are available from the Gene Expression Omnibus (GEO) repository under accession number GSE109414 (https://www.ncbi.nlm.nih.gov/geo/query/acc.cgi?acc=GSE109414). The authors declare that all other data supporting the findings of this study are available within the article and its supplementary information files.
